# Simple Patterned Nanofiber Scaffolds and Its Enhanced Performance in Immunoassay

**DOI:** 10.1371/journal.pone.0082888

**Published:** 2013-12-10

**Authors:** Jing Wang, Qin-shu Kang, Xiao-guang Lv, Jia Song, Na Zhan, Wei-guo Dong, Wei-hua Huang

**Affiliations:** 1 Department of Gastroenterology, Renmin Hospital of Wuhan University, Wuhan, China; 2 Key Laboratory of Analytical Chemistry for Biology and Medicine (Ministry of Education), College of Chemistry and Molecular Sciences, Wuhan University, Wuhan, China; 3 College of Science, Huazhong Agricultural University, Wuhan, China; 4 Department of Pathology, Renmin Hospital of Wuhan University, Wuhan, China; Taipei Medical University, Taiwan

## Abstract

Cancer has become the leading cause of death worldwide; early diagnosis and treatment of cancers is critical for the survival of the patients. The concentration of cancer markers in easy-to-access biological fluids can provide great assistance in screening for occult primary cancers, distinguishing malignant from benign findings, determining prognosis and prediction for cancer patients. The multiplex detection technology of a panel of cancer markers can greatly increase the accuracy of disease diagnosis. Herein, we briefly fabricate a high-throughput micro-immunoassay based on the electrospun polystyrene (PS) substrates to improve detection sensitivity. The immunoassay was evaluated by analyzing three different cancer biomarkers (AFP, CEA, VEGF). For AFP, CEA, VEGF immunofluorescence assay, the LOD of assay conducted on electrospun PS substrates before or after plasma and the conventional PS substrates were 0.42, 0.10, 1.12 ng/mL, 0.57, 0.09, 1.24 ng/mL, and 159.75, 26.19, 385.59 pg/mL, respectively (*P* < 0.05). Due to the high porosity and large surface area-to-volume ratio which is the foremost merit of nanostructures, and the plasma treatment which make the hydrophobic PS nanofibers hydropholic, the nanofibers substrates showed sufficient retention of immunoassay functionality and high potential for capture molecules immobilization. Consequently, the immunofluorescence assay conducted on electrospun PS substrates could significantly enhance the sensitivity and limits of detection.

## Introduction

Cancer is the first leading cause of death in developed countries and the second leading cause of death in developing countries [1]. Based on the GLOBOCAN 2008 estimates, about 12.7 million cancer cases and 7.6 million cancer deaths are estimated to have occurred in 2008 worldwide [2]. Moreover, the projections of estimated numbers of new cancer deaths may rise to more than 13.1 million in 2030. Early diagnosis and treatment of cancers is critical for the survival of the patients [3,4]. Both imaging and laboratory medicine are the efficient methods for malignant tumor screening; especially laboratory medicine plays a crucial role in screening out malignant tumors in an earlier stage [5,6]. Researches have shown that the concentration of cancer markers in easy-to-access biological fluids can provide great assistance in multiple clinical settings, including estimating risk of disease, screening for occult primary cancers, distinguishing malignant from benign findings, determining prognosis and prediction for cancer patients, and monitoring status of the disease [7–10]. There are numerous classical cancer markers used clinically, such as alpha fetoprotein (AFP) and carcinoembryonic antigen (CEA), meanwhile, increasing numbers of new cancer markers are being discovered, such as vascular endothelial growth factors (VEGF) [11]. Nonetheless, no single cancer marker is sensitive and specific enough to meet the stringent diagnostic criteria. Studies have shown that the combined measurement of a panel of cancer markers can greatly increase the accuracy of disease diagnosis [12,13]. Therefore, multiplex immunoassay technology in high-throughput detection is necessary and can be the direction of development of immunoassay technology in the near future.

As methods to detect the presence of cancer markers in biological fluids on the basis of specific reactions between antibodies and antigens, immunoassays have been widely used as primary tools for the diagnosis of cancers. However, there are some limitations for conventional immunoassays, which include the single target analyte test, the deficiency in quantifying minute amounts of biomolecules, the larger volume of samples and longer detection time required when the sample population is large or several targets need to be analyzed simultaneously in each sample [14,15]. What’s more, most immunoassay systems rely on two-dimensional surfaces of planar substrates for immobilizing capture molecules [16]. Nevertheless, immobilization of large amounts of capture molecules on substrates is extremely required in obtaining signal intensities sufficient to detect low abundance analytes in samples [17,18]. A rational approach for overcoming this would be to increase the substrate’s surface area. Electrospun polymeric nanofiber scaffolds that have high porosity and large surface area are attractive substrates for immobilizing capture molecules. The nanofibers for these applications can be fabricated by electrospinning with a broad range of chemically [19] and biologically [20] active surfaces, which is a technique to produce fibers from an electrically driven jet of polymeric fluid with a range of diameters from micrometers to nanometers [21]. Electrospinning is a versatile and cost-effective method for fabricating substrates consisted of a matrix of nanoscale fibers [22]. Due to their large area-to-volume ratio, electrospun nanofiber scaffolds can immobilize large numbers of capture molecules, which enhances the reactivity and sensitivity of immunoassay system [13]. For decades, electrospinning has been used to fabricate highly sensitive sensors with various applications, including optical [23], chemical [24], and biological sensing [25,26]. Otherwise, as an evolution of the planar microassay, electrospun nanofiber scaffolds can offer distinct advantages, including of simpler fabrication, less expense, faster binding kinetics, greater flexibility in array preparation, and superior detection sensitivity, which make it outstanding for immunoassay systems.

Herein, we delineate a high-throughput micro-immunoassay based on the electrospun polystyrene (PS) substrates, designed to improve detection sensitivity, compared with the conventional PS substrates. Three different cancer biomarkers (AFP, CEA, VEGF) were analyzed to compare the analytical performance of both assay formats.

## Experimental Section

### Materials and reagents

Polystyrene (PS) (MW 192000), tetrahydrofuran (THF), dimethylformamide (DMF), carbonate-bicarbonate buffer, 0.01 M phosphate buffered saline (PBS), bovine serum albumin (BSA) were purchased from Sigma-Aldrich (Milwaukee, WI, USA). 

### Antibody and antigen reagents

CEA: capture (rabbit polyclonal CEA antibody, Fitzgerald, 70R-10664), detector (mouse monoclonal CEA antibody, Fitzgerald, 10C-CR2014M2), protein (Fitzgerald, 30C-CP3012). AFP: capture (rabbit polyclonal AFP antibody, Fitzgerald, 70R-10676), detector (mouse monoclonal AFP antibody, Fitzgerald, 10-A100B), protein (Fitzgerald, 30-1029). VEGF: capture (affinity-purified polyclonal antibody, R&D Systems, AF-293-NA), detector (Rabbit highly purified VEGF antibody, Fitzgerald, 70R-13764), protein (Recombinant human VEGF 165, R&D Systems, 293-VE-010). DyLight 405 goat anti-mouse IgG antibody (405313), DyLight 488 goat anti-mouse IgG antibody (405310), DyLight 649 donkey anti-rabbit IgG antibody (406406) were purchased from BioLegend (San Diego, CA, USA). All of the antibodies and proteins were resuspended and stored according to the manufacturer’s specifications. The standard antigen proteins were diluted to a panel of needed gradient concentrations (AFP, CEA: 0, 10^-1^, 10°, 10^1^, 10^2^, 10^3^ ng/mL, VEGF: 0, 62.5, 125, 250, 500, 1000, 2000 pg/mL) before use.

### Preparation of nanofiber scaffolds

The electrospinning solution was prepared by stirring and dissolving a measured amount of PS in a mixture of THF and DMF (1:2, v/v) to form a 23% (w/v) solution. The solution was placed in an 78 °C convection oven over night and then loaded in a 5 mL syringe with a flat needle. The electrospinning conditions were optimized to fabricate uniform PS nanofibers. To electrospin PS nanofibers, a 15 kV positive direct current high-voltage was applied to the solution via the flat metal needle and a constant feeding rate of the polymer solution (0.5 mL/h) was provided by the syringe pump (Longer, China). To collect the electrospun nanofiber webs, either a grounded clean aluminium foil or a grounded glass slide was used. The distance between the collector and the needle tip was 15 cm. The electrospun PS nanofibers were collected for 10 minutes and maintained at room temperature. 

### Scanning electron microscopy (SEM)

To determine morphology, the PS nanofibers were examined by scanning electron microscopy (SEM; X-650, Hitachi, Tokyo, Japan). The stability of the electrospun PS substrate was evaluated by soaking in PBS for 4.0 h, washing with deionized water, and drying in a desiccator. Changes in morphology after soaking in the solution were examined by SEM. Image analysis software (Image J 1.41) was used to measure the electrospun fiber diameter.

### Direct-pattern AZ 5214 photoresist on nanofibers and UV exposure

A special designed fistulous module with a 2 mm diameter was used for the direct patterning of AZ 5214 positive photoresist (Electronic Materials USA, USA). Due to the unique properties of AZ 5214 photoresist, the uncured photoresist could penetrate into the porous nanofibers quickly, and then bond with the surrounding PS nanofibers and glass slide at the bottom. After direct patterning of AZ 5214, the nanofiber scaffold was exposed under to UV (300 W) for 30 s to cure the photoresist.

### Fluorescence immunoassay

After treated with plasma cleaner (Harrick Plasma, Ithaca, NY, USA) at lower level for 10 s, 2 μl capture antibody (20 μg/mL) was added to each well and soaked for 18-24 hours at 4 °C. After adsorption of antibody, nanofibers were washed with 0.01 M PBS washing buffer three times. The plasma treatment made the hydrophobic PS nanofibers hydropholic, facilitating infiltration of antibody or buffer solution through the nanofiber scaffolds. Washing of the resultant PS nanofibers could remove the unattached or weekly attached antibodies. Subsequently, passivation of blocking solution (1.0 wt% BSA in PBS solution) was used to make nonspecific binding minimized. Followed blocking was the soaking with different cancer marker antigens with gradient concentrations (1 h at 37 °C), the detect antibodies (1 h at 37 °C) and fluorescent antibodies (0.5 h at 37 °C) solution. Between each step three times of washing with PBS was performed. The complete fluorescence immunoassay procedure was repeated at least 5 times. The fluorescence images were obtained by an inverted fluorescence microscope (Axio Observer Z1, ZEISS, Germany) and the intensity of fluorescence signals was analyzed using the software Image J.

### Statistical analysis

All data were showed as the mean ± standard error of the mean, and then subjected to the independent sample *t*-test or one-way ANOVA. Statistical significance was defined as a value of *P* < 0.05.

## Results and Discussion

### Fabrication and evaluation of the micro-immunoassay

**Figure 1:**
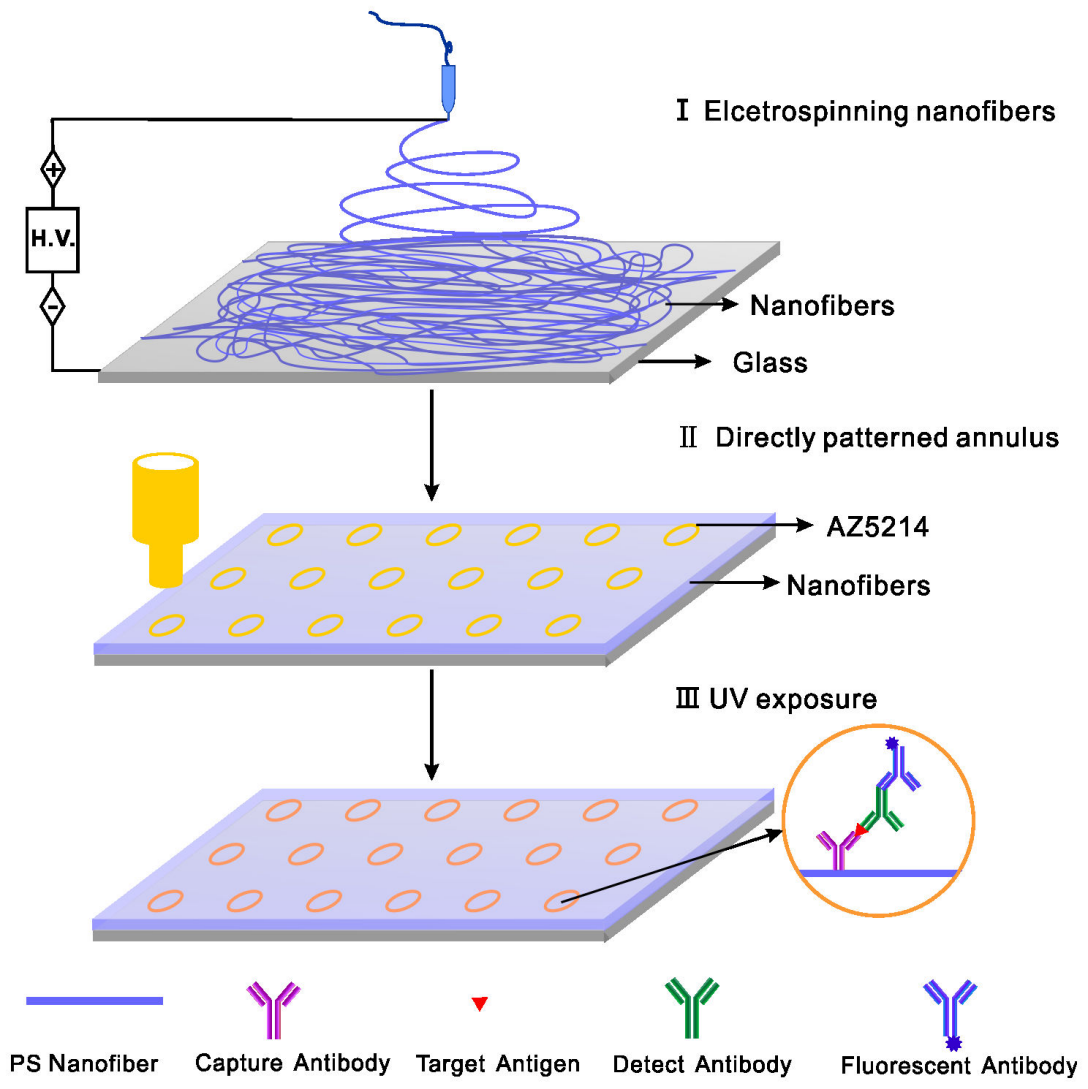
Schematic diagram of fabricating the micro-immunoassay with PS nanofibers substrate.

**Figure 2:**
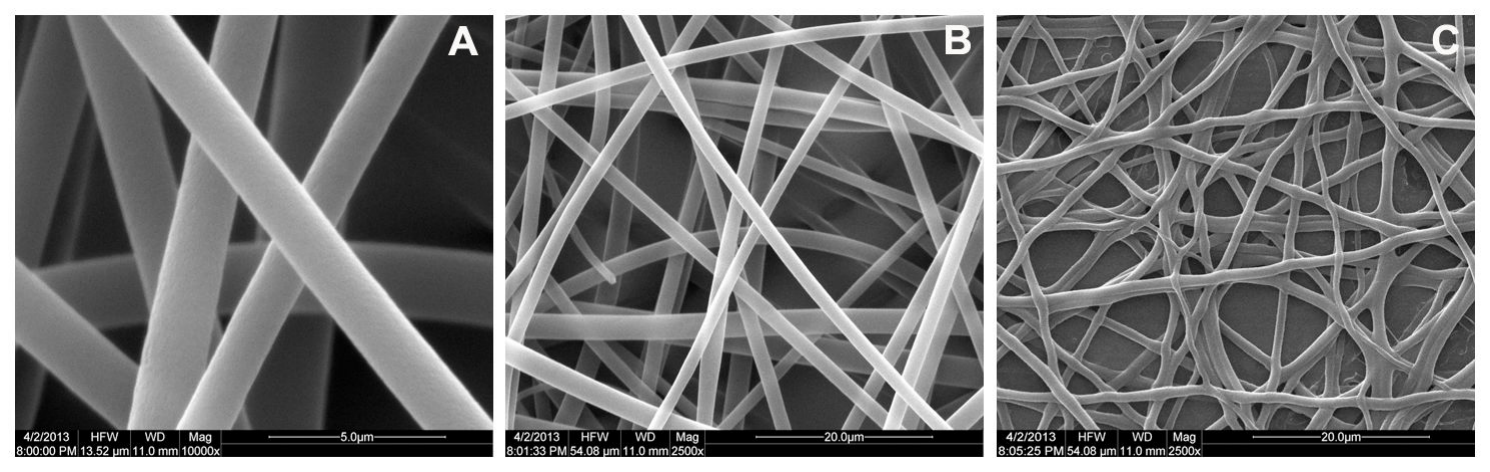
Morphologies of electrospun PS fibers examined by SEM. (A) Superfine fibers with a diameter of 1.0 μm (10000×). (B) and (C) PS fibers’ morphology before and after soaking with aqueous solution (2500×).

Overall procedure to prepare the micro-immunoassay with PS nanofibers was described in Figure 1. All of the electrospinning processes were performed under the same conditions, and the PS membrane was composed of ultrathin nanofibers of less than 1.0 μm in diameter (Figure 2). The thickness PS membrane could be controlled by the electrospinning time. After collecting for 10-12 min, we could obtain a membrane with a thickness about 50 μm, which was used in the study. 

As the PS substrate will be in contact with aqueous solution for a long period of time during the heterogeneous immunoassay, the stability of PS nanofibers in water should be taken into consideration. To confirm that the electrospun PS substrate can withstand the immunoassay processes of soaking in aqueous solution, the nanofibers were soaked in PBS for 4.0 hours and dried, then the morphology of the soaked fibers was examined by SEM. Figure 2 shows the SEM images of the PS fiber matrix before and after soaking with solution, and the overall structure and diameter was unchanged.

**Figure 3:**
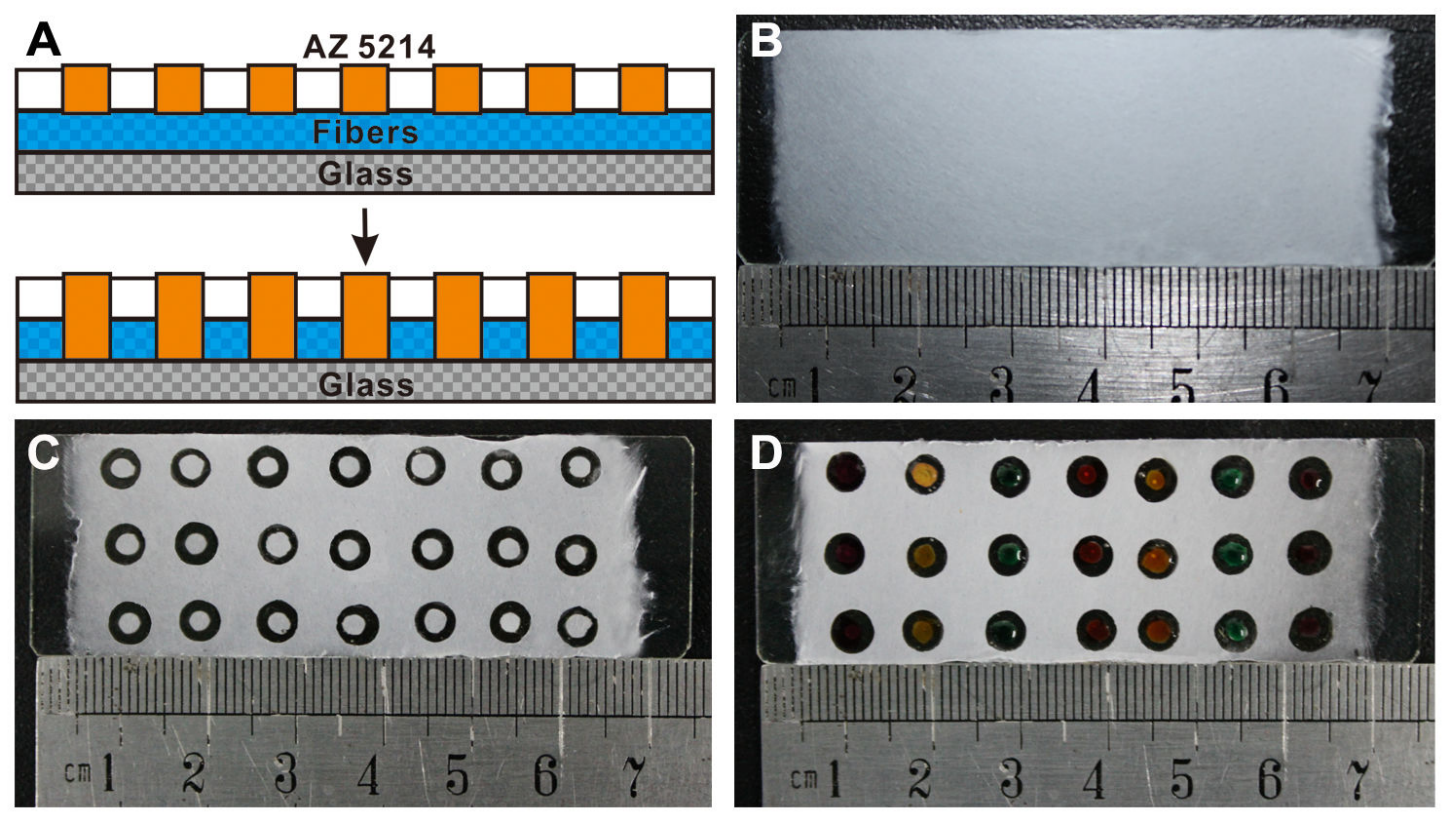
Illustration of the principle of using AZ 5214 to pattern the porous PS nanofiber scaffolds. (A) Schematic diagram of AZ 5214 penetrating through the porous structure, and bonding with the glass substrate and the surrounding fibers (cross section). (B) and (C) Digital camera pictures of the top view of real products. (D) Micro-chambers filled with different color inks.

In this work, AZ 5214 was chose to localize nanofibers substrates with different patterned configurations because of its low viscosity, rapid permeability, good adhesive and polymerization, and also due to its chemically inert and hydrophobic nature. These properties made it suitable to be good barriers to separate nanofibers into different diameter chambers. Figure 3A illustrated that the AZ 5214 photoresist directly patterned onto the surface of nanofibers can penetrate through the porous structure quickly, and bonds with the glass substrate and the surrounding fibers within a short time. Figure 3C and D showed a top view of the nanofibers substrates and micro-chambers filled with different color inks. It is showed to confirm that no leakage or diffusion of ink occurred during the whole soaking period.

### Capture antibody immobilization on electrospun PS substrates

The PS nanofibers before plasma are inherently bio-inert as the conventional PS substrates, as PS provides a hydrophobic surface. The air trapped in the nanofibers scaffolds can also impedes direct contact of solution and PS fibers. Treating with plasma cleaner for only 8-12 s can change the static contact angle (SCA) significantly from 140 ° to approx. 75 °, which is acceptable for capture molecules immobilization, for the solution can cover the entire substrate surface. 

**Figure 4:**
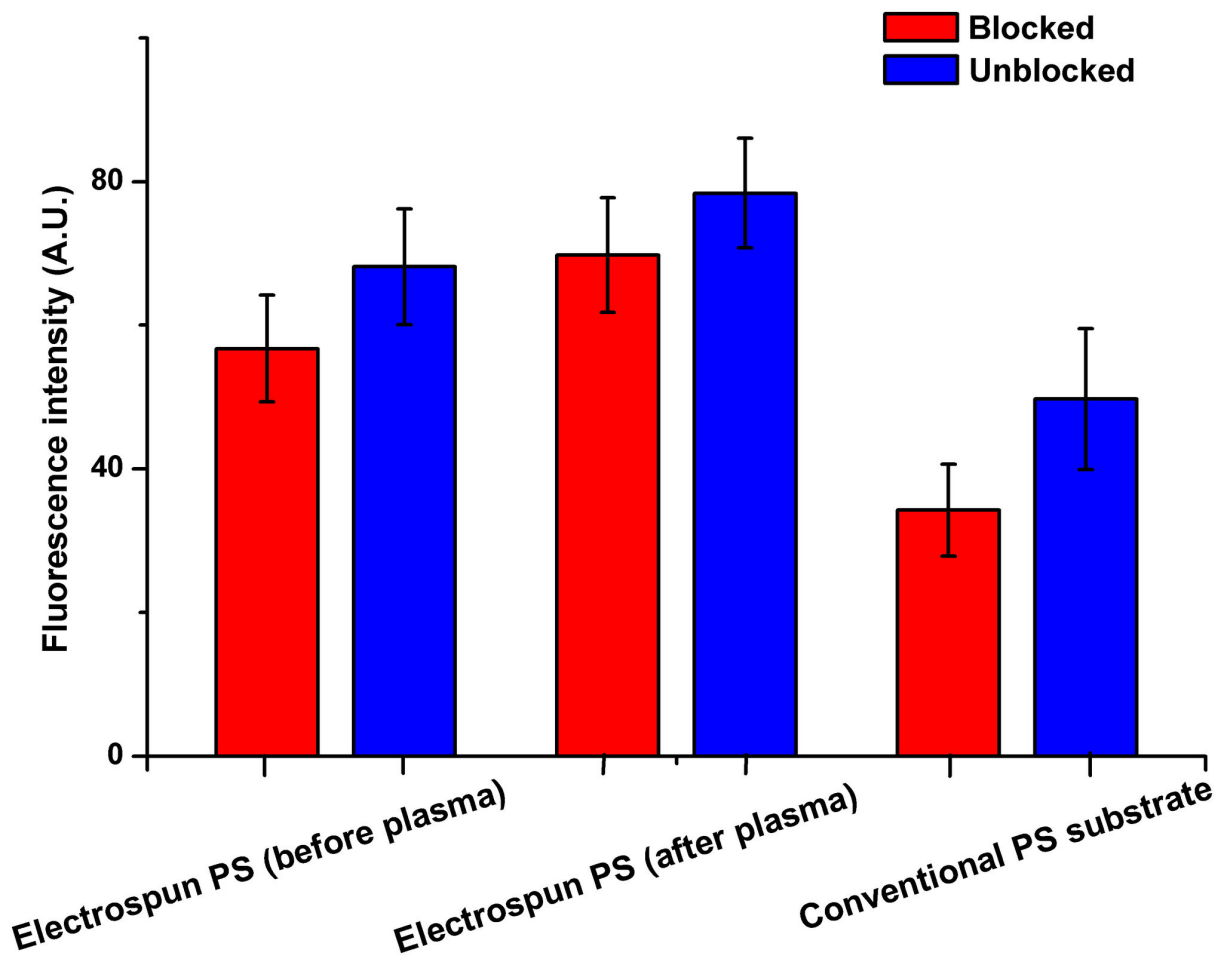
Comparison of fluorescence intensity obtained from different substrates with AFP antigen (100 ng/mL) between blocked and unblocked ones.

After immobilization of the capture antibody on the PS substrates, nonspecific binding should be inhibited or blocked by the BSA (1% w/v) regent. The nonbiofouling test was conducted on electrospun PS substrates before or after plasma and conventional PS substrate. The results shown in Figure 4 demonstrated that blocking reagents was essential for immunofluorescence assay run on both electrospun PS substrates and conventional PS substrates, though the differences in fluorescence intensity from the unblocked samples were 1.5-2.0 folds that of the blocked ones. The elevated signal in the case of the electrospun or conventional PS substrates can be explained as a consequence of nonspecific binding, which is a drawback of the biofouling on the hydrophobic substrates and unstable antibody immobilization. 

### Signal enhancement of sandwich immunoassay on electrospun PS substrates

**Figure 5:**
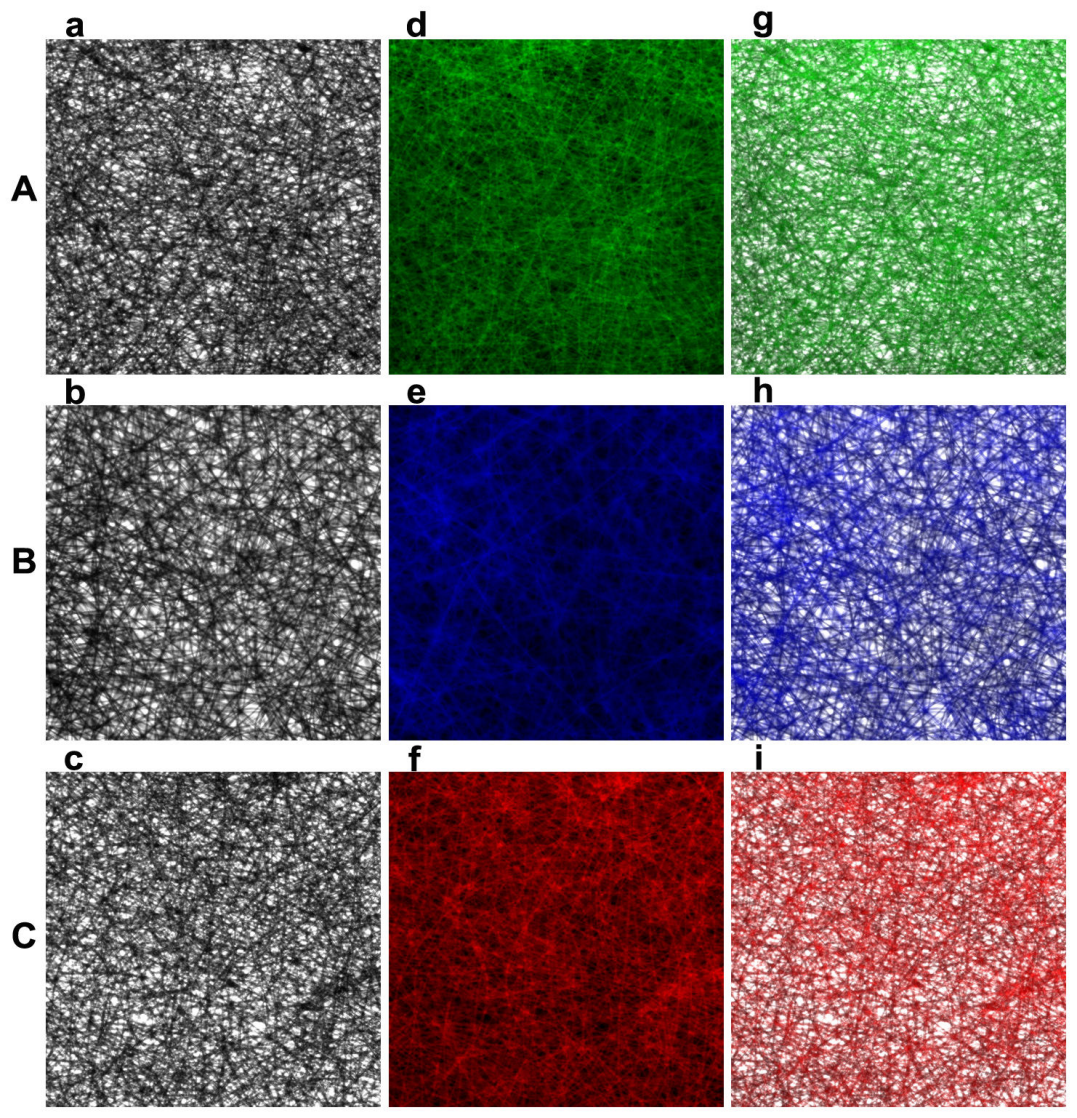
Fluorescence pictures of cancer biomarkers on electrospun PS substrates obtained by an inverted fluorescence microscope (200×). (A) AFP (DyLight 488, green), (B) CEA (DyLight 405, blue), (C) VEGF (DyLight 649, red); (a-c) light field, (d-f) fluorescence field, (g-i) superposition view of the two fields.

**Figure 6:**
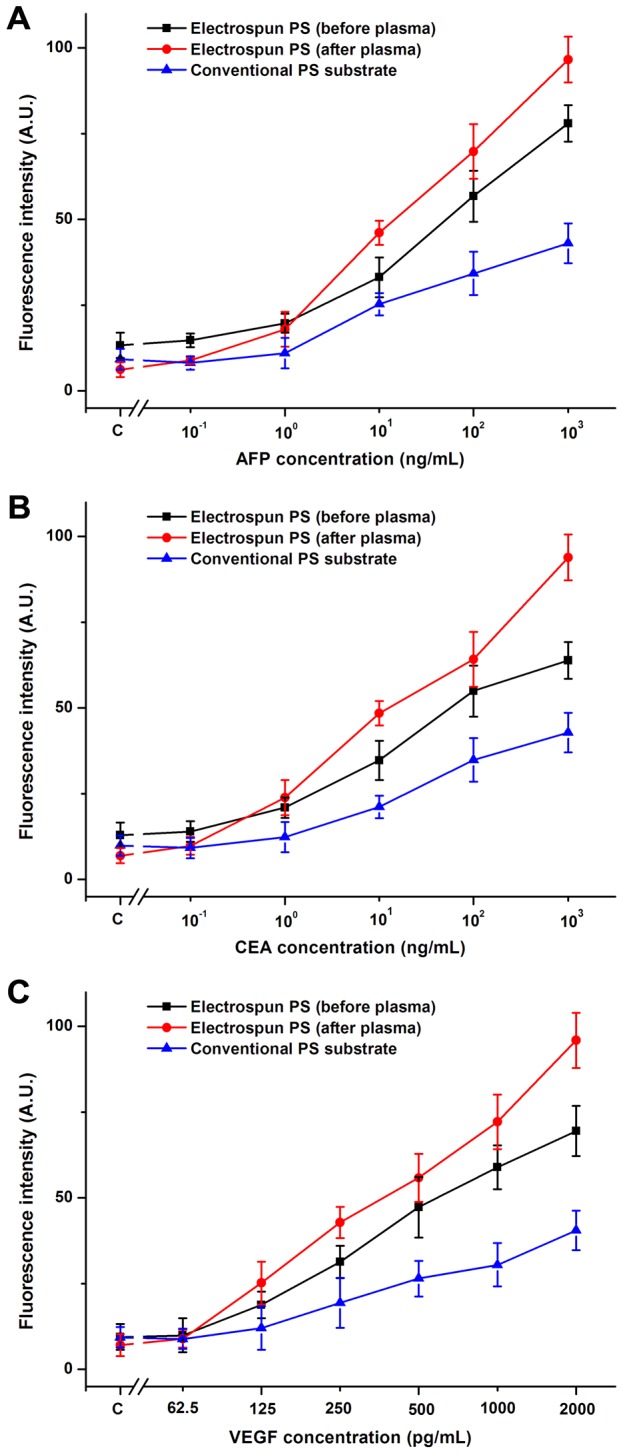
Relationship between fluorescence intensity and cancer marker concentration on different substrates.

Three different kinds of cancer biomarkers (AFP, CEA, VEGF) were used to compare the analytical performance and evaluate the feasibility and stability of both assay formats. The fluorescence pictures of each cancer biomarkers on electrospun PS substrates obtained by an inverted fluorescence microscope were shown in Figure 5. Followed the analyzing of the intensity of fluorescence signals using the software Imang J, the immunofluorescence assay calibration curves was drew up of the mean fluorescence intensity and the antigen concentration (Tables S1, S2, S3 in File S1). The calibration curves run on electrospun PS platforms before or after plasma and the conventional PS platforms with 99% confidential level are illustrated in Figure 6. Immunoassays on electrospun or conventional PS substrates both demonstrated a good relationship between the fluorescence intensity and antigen concentration. 

The limits of detection (LOD) of immunofluorescence assay on all substrates were calculated using the widely used formula, stated that fluorescence intensity corresponding to LOD equals to average fluorescence intensity of the blank samples plus triple standard deviation (μ+3σ) [14]. For AFP and CEA immunofluorescence assay, the LOD of assay conducted on electrospun PS substrates before or after plasma and the conventional PS substrates were 0.42, 0.10, 1.12 ng/mL and 0.57, 0.09, 1.24 ng/mL, respectively (*P* < 0.05). For VEGF immunofluorescence assay, the LOD of assay also had significant decrease from 385.59 pg/mL conducted on conventional substrates to 159.75 pg/mL on electrospun PS substrates before plasma and 26.19 pg/mL on electrospun PS substrates after plasma, respectively (*P* < 0.05). The differences of LOD among the three substrates are significant (*P* < 0.05), and the immunofluorescence assay conducted on the electrospun PS substrate after plasma has the lowest LOD and significantly enhanced sensitivity.

These phenomena could be explained as the merit of nanostructures, as larger surface area-to-volume increases possibility for biomolecules to contact with fibers substrates, thus the efficiency and sensitivity of electrospun PS substrates to detect low amount of analyte was higher than conventional planar PS substrates. When comparing between electrospun PS substrates before and after plasma, although both could provide equivalent detection range, the fluorescence intensity from the electrospun PS substrates after plasma exhibited the strongest signal. As explored, sensitivity and detection limits was further enhanced by covalently immobilizing antibodies. Usually, detection of cancer markers or other cellular metabolites in easy-to-access biological fluids is conducted using enzyme-linked immunosorbent assay (ELISA) in PS microwell plates due to the better sensitivity and detection limits than that of the fluorescence-based methods [27]. Although fluorescence-based methods are superior to ELISA in many aspects, such as the simple procedure and ease of multiplex detection simultaneously, the use for clinical applications has been limited due to its poor performance, as shown in this research. However, the high-throughput micro-immunoassay based on the electrospun PS substrates reported here has significant advantages, including of simpler fabrication, less reagent needed, much cheaper, greater flexibility in array preparation, and superior detection sensitivity. So, we anticipate that using electrospun nanofibers substrates could overcome the limitation of fluorescence-based detection and would be outstanding for immunoassay system.

## Conclusions

In this research, we fabricated a high-throughput micro-immunoassay with PS nanofibers and evaluated its performance in practical application. Three different cancer biomarkers (AFP, CEA, VEGF) were used to compare the analytical performance and evaluate the efficiency, feasibility, sensitivity, and stability of electrospun PS substrates before or after plasma compared with the conventional PS substrates. Due to the high porosity and large surface area-to-volume ratio which is the foremost merit of nanostructures, and the plasma treatment which make the hydrophobic PS nanofibers hydropholic, the nanofibers substrates showed sufficient retention of immunoassay functionality and high potential for capture molecules immobilization. Consequently, the immunofluorescence assay conducted on electrospun PS substrates significantly enhanced the sensitivity and limits of detection.

## Supporting Information

File S1 **Table S1, Relationship between fluorescence intensity and AFP concentration on different substrates.** Table S2, Relationship between fluorescence intensity and CEA concentration on different substrates. Table S3, Relationship between fluorescence intensity and VEGF concentration on different substrates.
(DOCX)
