# Supplementary material for: Simple Patterned Nanofiber Scaffolds and Its Enhanced Performance in Immunoassay
Source: PLoS One. 2013 Dec 10;8(12):e82888. doi: 10.1371/journal.pone.0082888 (PMC3858307; doi:10.1371/journal.pone.0082888)
Supplement: File S1 — Table S1, Relationship between fluorescence intensity and AFP concentration on different substrates. Table S2, Relationship between fluorescence intensity and CEA concentration on different substrates. Table S3, Relationship between fluorescence intensity and VEGF concentration on different substrates. (DOCX) [file pone.0082888.s001.docx]

**Supplemental Results**

**Table S1 Relationship between fluorescence intensity and AFP concentration on different substrates.**

| AFP concentration (ng/mL) | Fluorescence intensity (A.U.) (mean ± standard deviation) | | |
| --- | --- | --- | --- |
|  | Electrospun PS (before plasma) | Electrospun PS  (after plasma) | Conventional PS substrate |
| 0 | 13.32828±3.69381 | 6.22043±6.22043 | 9.24832±2.97132 |
| 10^-1^ | 14.76205±2.00424 | 8.92277±1.02585 | 8.15777±1.93745 |
| 10^0^ | 19.72933±2.74839 | 18.0095±5.13289 | 11.02836±4.37242 |
| 10^1^ | 33.14701±5.70339 | 46.1075±3.54629 | 25.28463±3.28346 |
| 10^2^ | 56.76667±7.42495 | 69.8005±7.99066 | 34.27548±6.37362 |
| 10^3^ | 78.04128±5.31946 | 96.6115±6.68661 | 43.06252±5.78263 |

**Table S2 Relationship between fluorescence intensity and CEA concentration on different substrates.**

| CEA concentration (ng/mL) | Fluorescence intensity (A.U.) (mean ± standard deviation) | | |
| --- | --- | --- | --- |
|  | Electrospun PS (before plasma) | Electrospun PS  (after plasma) | Conventional PS substrate |
| 0 | 12.94828±3.81028 | 6.93816±2.19326 | 9.89236±3.10282 |
| 10^-1^ | 13.95735±3.17356 | 9.84362±2.62595 | 9.19829±2.93715 |
| 10^0^ | 20.94718±2.90912 | 23.84736±4.98261 | 12.38726±4.28456 |
| 10^1^ | 34.72516±5.74761 | 48.48463±3.60126 | 21.14873±3.49227 |
| 10^2^ | 54.92733±6.03829 | 64.21862±8.19203 | 34.84726±6.29103 |
| 10^3^ | 63.87261±6.00218 | 93.95726±6.51526 | 42.83473±5.72816 |

**Table S3 Relationship between fluorescence intensity and VEGF concentration on different substrates.**

| VEGF concentration (pg/mL) | Fluorescence intensity (A.U.) (mean ± standard deviation) | | |
| --- | --- | --- | --- |
|  | Electrospun PS (before plasma) | Electrospun PS  (after plasma) | Conventional PS substrate |
| 0 | 9.47672±3.71938 | 7.08372±3.09371 | 9.35172±2.81937 |
| 62.5 | 9.94827±5.01837 | 8.98275±2.71630 | 8.90137±2.98163 |
| 125 | 18.82615±3.78491 | 25.19387±6.31038 | 12.09217±6.20481 |
| 250 | 31.28636±4.81030 | 42.84715±4.49183 | 19.43782±7.10394 |
| 500 | 47.27361±8.45374 | 55.83713±7.01983 | 26.48216±5.30183 |
| 1000 | 58.92716±6.10437 | 72.18373±8.18373 | 30.49318±6.31183 |
| 2000 | 69.52162±7.01937 | 95.93872±7.92672 | 40.49862±5.91836 |
